# The Non-Classical MAP Kinase ERK3 Controls T Cell Activation

**DOI:** 10.1371/journal.pone.0086681

**Published:** 2014-01-27

**Authors:** Miriam Marquis, Salix Boulet, Simon Mathien, Justine Rousseau, Paméla Thébault, Jean-François Daudelin, Julie Rooney, Benjamin Turgeon, Claudine Beauchamp, Sylvain Meloche, Nathalie Labrecque

**Affiliations:** 1 Maisonneuve-Rosemont Hospital Research Centre, Montreal, Quebec, Canada; 2 Department of Microbiology, Infectiology and Immunology, University of Montreal, Quebec, Canada; 3 Department of Pharmacology and Molecular Biology, University of Montreal, Quebec, Canada; 4 Department of Medicine, University of Montreal, Quebec, Canada; 5 Institute of Research in Immunology and Cancer, University of Montreal, Quebec, Canada; Oklahoma Medical Research Foundation, United States of America

## Abstract

The classical mitogen-activated protein kinases (MAPKs) ERK1 and ERK2 are activated upon stimulation of cells with a broad range of extracellular signals (including antigens) allowing cellular responses to occur. ERK3 is an atypical member of the MAPK family with highest homology to ERK1/2. Therefore, we evaluated the role of ERK3 in mature T cell response. Mouse resting T cells do not transcribe ERK3 but its expression is induced in both CD4^+^ and CD8^+^ T cells following T cell receptor (TCR)-induced T cell activation. This induction of ERK3 expression in T lymphocytes requires activation of the classical MAPK ERK1 and ERK2. Moreover, ERK3 protein is phosphorylated and associates with MK5 in activated primary T cells. We show that ERK3-deficient T cells have a decreased proliferation rate and are impaired in cytokine secretion following *in vitro* stimulation with low dose of anti-CD3 antibodies. Our findings identify the atypical MAPK ERK3 as a new and important regulator of TCR-induced T cell activation.

## Introduction

The MAPKs ERK1 and ERK2 are activated upon stimulation of cells with a broad range of extracellular signals including antigens (Ags) [1], [2]. Activated ERK1/2 translocate to the nucleus to mediate the phosphorylation of transcription factors allowing cellular responses to occur [3]. The ERK1/2 MAPKs are rapidly phosphorylated in T cells following TCR activation. Interestingly, ERK1 is dispensable for CD8^+^ T cell proliferation following TCR engagement while ERK2 is necessary [4]. More recently, other members of the ERK family have been described [5] but their roles in T cell responses have not been described yet. ERK3 is another member of the MAPK family with highest homology to ERK1/2 [5], [6]. ERK3, and its paralogous protein ERK4, is considered an atypical MAPK since it lacks the conserved Thr-Xaa-Tyr motif in the activation loop and possesses a long C-terminal extension [5], [6]. The signaling events leading to ERK3 activation and its substrates or partners are still largely unknown. ERK3 is constitutively phosphorylated by group I p-21-activated kinases [7], [8] in resting cells and its phophorylation status does not change in response to various extracellular signals [9]. Contrary to ERK1/2, ERK3 has a very short half-life in exponentially proliferating cells [10], [11] and its half-life increases during differentiation processes that are coupled to cell cycle arrest [11]. Notably, overexpression of a stable form of ERK3 inhibits S phase entry in fibroblasts [11]. This suggests a possible role for ERK3 accumulation in cellular differentiation events.

Little is known about the physiological functions of ERK3. Genetic ablation of the *Erk3* gene has revealed that ERK3 plays an important role in fetal growth and lung maturation [12]. Recently, it was shown that ERK3 interacts with MK5 [13], [14]. This interaction leads to the phosphorylation and activation of MK5 and to the exclusion of both ERK3 and MK5 from the nucleus [13], [14]. Although ERK3 regulates MK5 activity, ERK3 ablation in HeLa cells and mouse embryonic fibroblasts only reduces MK5 activity by 50% [14]. The remaining MK5 activity is due to the fact that the close paralog of ERK3, ERK4, is also a physiological activator of MK5 [15], [16]. Unfortunately, the identification of MK5 as a binding partner of ERK3 did not provide any insight into the biological role of ERK3 since the function of MK5 is still unresolved [17], [18].

Naive T cells (CD44^lo^CD62L^hi^) circulate between lymphoid organs to patrol for the presence of invaders. The recognition of a foreign Ag presented by specialized Ag-presenting cells (APCs) in lymphoid organs leads to T cell activation. This activation is mediated by a cascade of intracellular signaling events following the interaction of the TCR/CD3 complex and CD4/CD8 co-receptors with peptide-MHC complexes [19]. Briefly, the Src kinase Lck (associated with CD4/CD8) phosphorylates the ITAM motifs contained in the intracellular portion of the CD3 chains. This recruits the ZAP-70 tyrosine kinase, which then becomes available for phosphorylation by Lck. This phosphorylation activates ZAP-70 that in turn phosphorylates different adaptor molecules (LAT, SLP-76). These adaptors then propagate the signal to three main pathways: ERK1/2, PLCγ1 (calcineurin and PKC) and the PI3K pathways. The engagement of these effector pathways leads to the regulation and activation of transcription factors that control gene expression leading to full activation, proliferation and differentiation of T cells. This expansion increases by up to 5000-fold the number of cells bearing an appropriate TCR. The activation and proliferation of T cells are accompanied by changes in their migration properties (able to migrate to the site of infection) and by their expression of effector functions (cytokine secretion or killing) allowing them to eliminate the infectious agent.

The classical MAPKs ERK1 and ERK2 play essential roles in TCR signaling following Ag recognition. ERK1 and ERK2 signaling trigger biochemical responses allowing T cell proliferation and differentiation [1], [2], [19]. Moreover, it was recently shown that ERK2, but not ERK1, is required for optimal CD8^+^ T cell proliferation and survival [4]. However, the expression profile and the role of the non-classical MAPKs, such as ERK3 and ERK4, have not been studied in T cells. Therefore, given the possible link of ERK3 with cellular differentiation, we studied its role in T cell activation, which requires concomitant proliferation and differentiation. Our results show that ERK3 expression is induced in both CD4^+^ and CD8^+^ T cells following T cell activation suggesting a possible role for ERK3 in T cell response. This induction of ERK3 is specific to TCR signaling and depends upon activation of the classical MAPKs ERK1 and ERK2. Importantly, ERK3-deficient T cells show a decrease in cell proliferation rate and cytokine secretion following *in vitro* anti-CD3 stimulation. In conclusion, the atypical MAPK ERK3 is a new and important player controlling TCR-induced T cell activation.

## Materials and Methods

### Mice

*Erk3* heterozygote mice [12] were bred under specific pathogen free (SPF) conditions at the Maisonneuve-Rosemont Hospital Research Centre. *Erk4*-GFP knock-in (*Erk4* ^ki/ki^) mice [20] were bred at Institute of Research in Immunology and Cancer under SPF conditions. B6129F1 were purchased from Taconic (Hudson, NY, USA). All animal experimental procedures were done according to the rules of Canadian Council on Animal Care and the protocol was approved by the Committee for the Protection of Animals of the Maisonneuve-Rosemont Research Center (# 2007–27 and 2011–27).

### Hematopoietic Chimeras

Fetal liver hematopoietic chimeras were generated as described [21]. Briefly, fetal liver was harvested from day 13.5 embryos and culture in RPMI supplemented with 5% FBS and 5% penicillin-streptomycin at 37°C until the genotyping was done. Fetal liver were gently dissociated in media, washed 2 times and re-suspended in PBS. 2×10^6^ fetal liver cells were injected i.v. into lethally irradiated (12 Gy) syngenic B6129F1 mice (5–7 wks old). Hematopoietic chimeras were analyzed 6–8 wks after grafting. The successful engraftment of *Erk3* ^−/−^ fetal liver cells was evaluated using the β-galactosidase reporter and was always 100%.

### Antibodies and Flow Cytometry

The following antibodies (Abs) were used: anti-CD4 (RM4-5), anti-CD8 (53–6.7), anti-CD3 (145-2C11), anti-CD44 (IM7), anti-TCRγδ (UC7-13D5), anti-CD62L (Mel-14), anti-TCR Vα8.3 (B21.14), anti-CD28 (37.51), anti-TGF-β1 (TW7-16B4) and anti-IL-10 (JES5-16E3) were purchased from Biolegend (San Diego, CA, USA); anti-CD4 (CL012PE and CL013F), anti-B220 (RA3-6B2), anti-CD69 (CL8969F), anti-CD25 (CL8925F and PC615.3) and anti-IgM were purchased from Cedarlane (Burlington, ON, Canada); anti-CD8 (53–6.7), anti-FoxP3 (MF23), anti-TNF-α (MP6-XT22), anti-TCR Vα3.2 (RR3-16), anti-TCR Vα11.1 (RR8-1) and anti-TCR Vβ screening panel were purchased from BD Biosciences (Mississauga, ON, Canada). Finally anti-TCR Vα2 (B20.1) was purchased from Life technologies (Burlington, ON, Canada). Cell stainings were performed as described previously [22].

### β-galactosidase Staining

Fluorescein digalactopyranoside (FDG; Sigma-Aldrich, Oakville, ON, Canada) staining was done as described by Chan et al. [23]. Briefly, 4×10^6^ cells were surface stained and re-suspended in 120 µl of PBS. Cells and diluted FDG (7.5 mM) were incubated 5 min at 37°C and 80 µl of warmed FDG was added to cells while gently vortexing. The reaction was stopped by adding 2 ml of ice-cold PBS and cells were kept on ice for 5 min. After centrifugation, cells were re-suspended in PBS 10% horse serum. Cells were then transferred to a 15°C water bath for 15–20 min to enhance β-galactosidase activity before cytometry analysis.

### T cell Stimulation

Splenocytes (2×10^6^) were stimulated in 24-well plates coated with different concentrations (0–3 µg/ml) of anti-CD3 Ab (145-2C11; Cedarlane) in complete RPMI (supplemented with 10% FBS, penicilin-streptomycin, 2-ME, sodium pyruvate and non-essential amino acids). After 12 to 72 h, cells were harvested and stained or re-stimulated for the analysis of cytokine production. When indicated, splenocytes were labeled with CFSE (Life technologies) as described previously [24]. In addition, some assays contained the MEK1/2 inhibitor U0126 (10 µM) (New England Biolabs, Whitby, ON, Canada). In some experiments, 5 µg/ml of soluble anti-CD28 (Cedarlane) was added to the stimulation.

### Cytokine Production

Splenocytes were activated *in vitro* with anti-CD3 Ab (Cedarlane) and harvested at 72 h. Activated splenocytes (10^6^ cells/ml) were stimulated with PMA (50 ng/ml) and ionomycin (500 ng/ml) (Sigma-Aldrich) in complete RPMI for 4 h at 37°C. Brefeldin A (10 µg/ml) (Sigma-Aldrich) was added for the last 2 h of culture. Intracellular cytokine detection was performed as described previously [24].

### Annexin V (AnV) Staining

After *in vitro* stimulation, cells were stained with anti-CD4 and anti-CD8 Abs followed by AnV and 7-AAD (BD biosciences) staining in AnV binding buffer (10 mM Hepes, pH7.4; 140 mM NaCl and 2.5 mM CaCl_2_).

### Immunoprecipitation and Immunoblotting

Cell lysis, immunoprecipitation, and immunoblot analysis were performed as previously described [11], [25], [26]. For immunoprecipitation experiments, 600 µg of total proteins extracted from total splenocytes stimulated for 72 h with anti-CD3 Ab (80% of the cells are T cells after stimulation) or unstimulated were incubated with polyclonal rabbit anti-HA (sc-805, Santa Cruz Biotechnology, Dallas, TX, USA) or polyclonal rabbit anti-ERK3 [11] Abs for 2 h at 4°C. Protein A-agarose beads were added for an additional 4 h at 4°C and the beads were washed four times in lysis buffer. Immunoprecipitated proteins complexes and whole cell extracts were analyzed by immunoblotting with rabbit monoclonal anti-ERK3 (Epitomics, Burlingame, CA, USA), mouse monoclonal anti-MK5 (sc-46667, Santa Cruz biotechnology) and mouse monoclonal anti-α-tubulin (Sigma-Aldrich). For phospho-Ser189 ERK3 analysis, 110 µg whole cell extract of splenocytes stimulated for 72 h with anti-CD3 Ab or unstimulated were analyzed by immunoblotting with polyclonal anti-phospho-ERK3(Ser-189) [11] and rabbit monoclonal anti-ERK3 (Epitomics). For cyclin D3 and Cdc14A levels analysis, 50 µg whole cell extract of splenocytes stimulated for 72 h with anti-CD3 Ab or unstimulated form wild-type or *Erk3* ^−/−^ hematopoietic chimeras were analyzed by immunoblotting with rabbit monoclonal anti-ERK3, mouse monoclonal anti-Cdc14A (R&D Systems, Minneapolis, MN, USA) and rabbit polyclonal anti-cyclin D3 (sc-182, Santa Cruz Biotechnology).

### RT-PCR and Quantitative RT-PCR

RNA from anti-CD3 stimulated T cells was extracted using Trizol (Life technologies) according to the manufacturer’s instructions. cDNA was synthesized using Superscript RT and synthetic oligo(dT) (Life technologies). Conventional PCR was performed with mouse *Erk4* primers: forward, 5′-GCGCAAGCTGCTCCCGGACGTC-3′, and reverse, 5′-GCCGCCTGCTTCCAGTGCGACAG-3′. *Gapdh* primers for conventional PCR were: forward, 5′-CTACAGCAACAGGGTGGTGG-3′, and reverse, 5′-TATGGGGGTCTGGGATGG-3′. HPRT primers were described previously [27]. For the quantification of *Erk3* mRNA level by quantitative RT-PCR (qRT-PCR), mouse *Erk3* and *Gapdh* primers and probes were purchased from Applied Biosystems (Burlington, ON, Canada) (Taqman gene expression assay ID: *Erk3 = * Mm00727050_s1; *Gapdh* =  Mm99999915_g1). Each reaction was performed in triplicate using a real-time cycler ABI Prism 7500 (Applied Biosystems). The ΔCt value for each sample was determined by calculating the difference between the Ct value of the target and the Ct value of the endogenous reference gene (*Gapdh*). Then, the ΔΔCt value for each sample was determined by subtracting the mean of ΔCt value of the sample from the ΔCt value of a reference sample as described earlier [28]. The relative level of the target gene expression was calculated using 2^−ΔΔCt^.

## Results

### Inducible ERK3 Expression in T Lymphocytes

**Figure 1:**
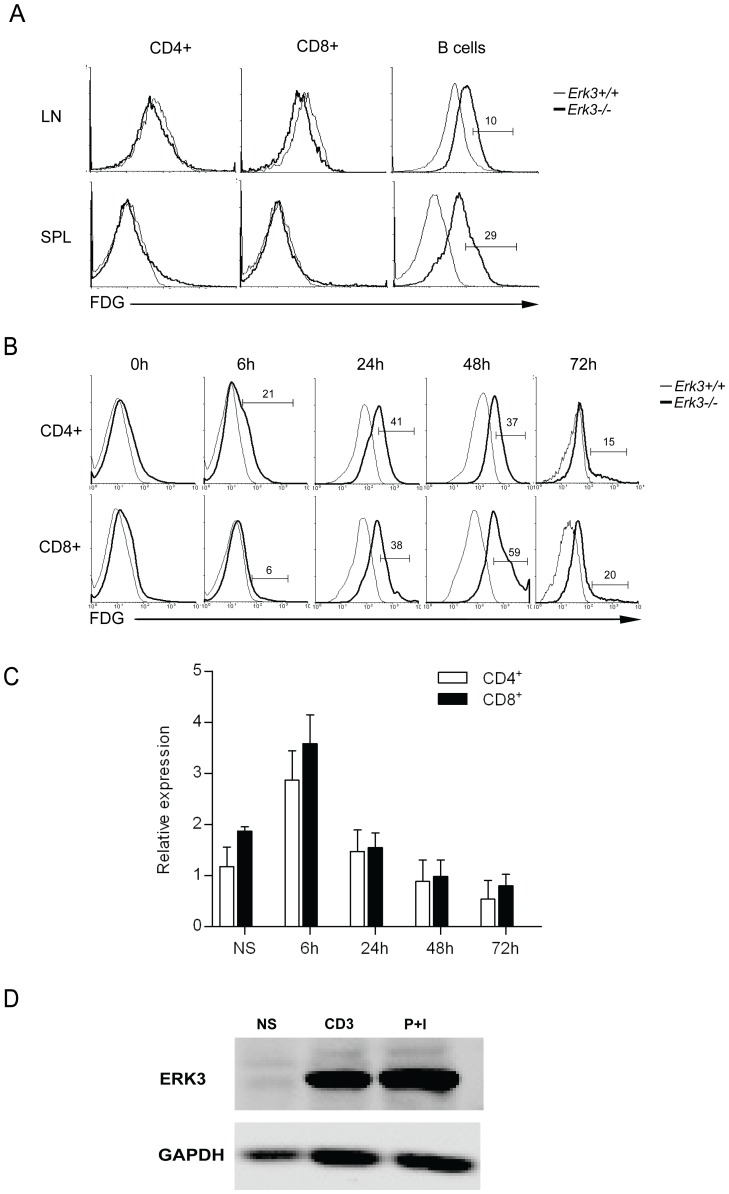
ERK3 expression is induced following activation of T lymphocytes. *A*, Resting T lymphocytes do not express ERK3. Splenocytes (SPL) and LN cells from *Erk3* ^+/+^ and *Erk3* ^+/−^ mice were stained with anti-CD4, anti-CD8 and anti-B220 Abs followed by intracellular detection of β-galactosidase activity using FDG. FDG staining is shown for CD4^+^ T cells, CD8^+^ T cells and B cells. *Erk3* ^+/+^ cells were used as a negative control for FDG staining. The data are representative of at least 3 independent experiments. *B*, Induction of ERK3 expression by T cells following anti-CD3 stimulation. Splenocytes from *Erk3* ^+/+^ and *Erk3* ^+/−^ mice were *in vitro* stimulated with coated anti-CD3 Abs for 6, 24, 48 and 72 h. Cells were cell surface stained with anti-CD4 and anti-CD8 Abs followed by FDG staining to measure β-galactosidase activity. The overlays show β-galactosidase activity (FDG) in CD4^+^ (top) and CD8^+^ (bottom) T cells at different times after activation. *Erk3* ^+/+^ splenocytes were used as negative control for FDG staining. The data are representative of four independent experiments. *C*, *Erk3* mRNA expression by sorted CD4^+^ and CD8^+^ T cells after anti-CD3 stimulation. Splenocytes from wild-type mice were either unstimulated (NS) or stimulated with coated anti-CD3 Ab (3 µg/ml) for 6, 24, 48 and 72 h followed by cell sorting. *Erk3* transcription was measured by qRT-PCR and normalized to *Gapdh*. Data are presented as a relative expression to a reference sample. *D,* ERK3 protein is detectable 48 h after T cell activation. Western blot analysis of ERK3 protein in lysates of unstimulated (NS), anti-CD3 stimulated (α-CD3) and PMA-ionomycin (P+I) stimulated wild-type splenocytes. Anti-GAPDH Ab was used as loading control.

We first evaluated if ERK3 was expressed in peripheral T cells. To do so, we used a knock-in mouse model in which the coding sequence of *Erk3* is replaced by a β-galactosidase reporter [12]. As shown in Fig. 1*A*, using a specific fluorescent substrate of β-galactosidase (FDG), we could not detect any significant β-galactosidase activity in resting CD4^+^ and CD8^+^ T lymphocytes from the spleen (SPL) and lymph nodes (LNs) of *Erk3* ^+/−^ mice suggesting that the *Erk3* gene is not transcribed in these cells. However, we could easily detect *Erk3* transcription in peripheral B cells (Fig. 1*A*). Further analysis failed to detect any FDG staining in specific subset of T cells such as naive and activated/memory T cells (data not shown). We then tested if stimulation of T cells via their TCR leads to induction of *Erk3* transcription. As shown in Fig. 1*B*, stimulation of T cells with anti-CD3 Abs induced *Erk3* transcription in both CD4^+^ and CD8^+^ T cells as detected by FDG staining. The transcriptional activation of the *Erk3* locus was transient, being detected as early as 6 h after T cell activation and almost completely extinguished after 72 h in both CD4^+^ and CD8^+^ T lymphocytes (Fig. 1*B*). Similar results were obtained using PMA and ionomycin stimulation (data not shown). Since the FDG staining assay relies on the detection of the activity of the β-galactosidase reporter and thus requires sufficient accumulation of the β-galactosidase protein, we directly evaluated the transcription of the endogenous *Erk3* gene by qRT-PCR. As shown in Fig. 1*C*, *Erk3* mRNA levels quickly raised following anti-CD3 stimulation and returned to background level at 48 h. The fact that the peak of *Erk3* mRNA accumulation differs from the peak of FDG staining probably reflects the need to accumulate enough β-galactosidase protein before being able to detect its activity. Moreover, the high stability of the β-galactosidase protein also allows the detection of FDG staining at time points where the *Erk3* locus is less transcribed. Since the ERK3 protein has been reported to be unstable in proliferating cell lines [11], we next verified if we could detect ERK3 protein expression in activated T cells that are actively dividing. The expression of ERK3 protein was detected by western blot only in T cells activated for 48 h with anti-CD3 Abs or PMA plus ionomycin (Fig. 1*D*).

### The ERK3 Protein is Phosphorylated and Associates with MK5 in T cells

**Figure 2:**
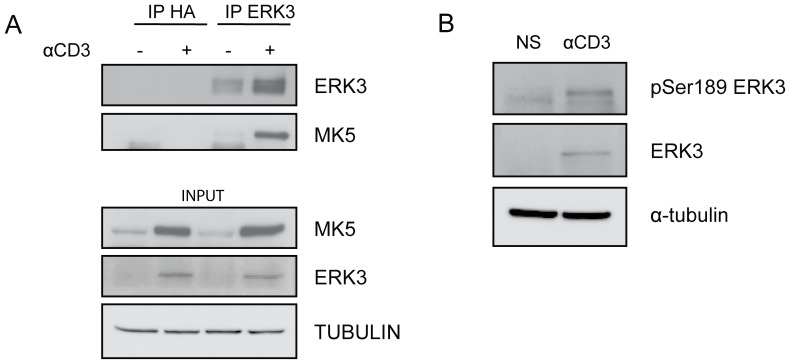
ERK3 is phosphorylated and associates with MK5 in activated primary T cells. *A*, ERK3 interacts with MK5 in activated T cells. Anti-CD3 stimulated (+) or unstimulated (-) total splenocytes from WT mice were collected 72 h after stimulation. Cells were lysed and protein complexes were immunoprecipitated (IP) with rabbit polyclonal anti-ERK3 or rabbit polyclonal anti-HA Abs as indicated. Total lysates (INPUT) and immunoprecipitated proteins were analysed by immunobloting with anti-ERK3, anti-MK5 and anti-tubulin Abs. *B*, ERK3 Ser189 is phosphorylated in activated T cells. Anti-CD3 stimulated or unstimulated (NS) total splenocytes from WT mice were collected 72 h after stimulation. Cells were lysed and proteins were analyzed by immunobloting with anti-ERK3, anti-pSer189 ERK3 and anti-tubulin Abs. The results shown are representative of three independent experiments.

We then evaluated whether ERK3 also associates with MK5 in primary T cells. As shown in Fig. 2*A*, MK5 was co-immunoprecipitated with ERK3 in activated T cells. This indicates that MK5 is also a physiological binding partner of ERK3 in primary T lymphocytes. Interestingly, we observed that, similar to ERK3, expression of MK5 is also up-regulated upon T cell activation. ERK3 phosphorylation was shown to be necessary for its association with MK5 [9]. In agreement with this, we found that ERK3 is phosphorylated on Ser189 in activated T cells (Fig. 2*B*). This suggests that the ERK3 signaling pathway is fully functional in T cells.

### The Classical MAPKs ERK1 and ERK2 Regulate ERK3 Expression in T Lymphocytes

**Figure 3:**
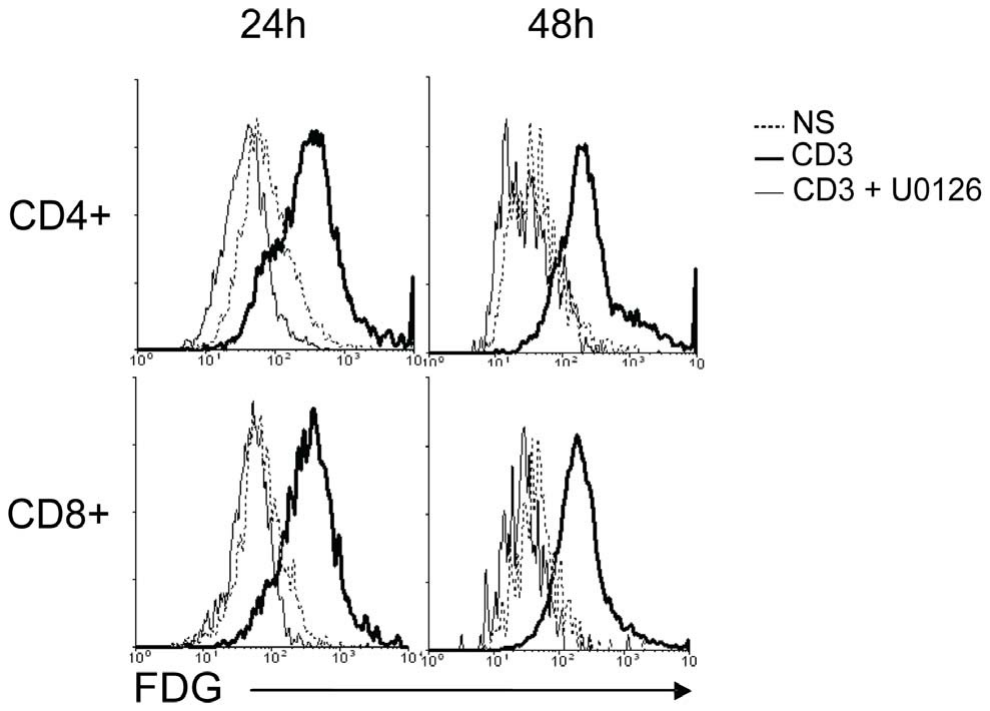
*Erk3* transcription is controlled by the classical ERK1/2 MAPK pathway. Splenocytes from *Erk3* ^+/−^ mice were stimulated with coated anti-CD3 Abs (1 µg/ml) for 24 h or 48 h in the presence or absence of the selective pharmacologic inhibitor of MEK1/2, U0126. Cells were cell surface stained with anti-CD4 and anti-CD8 Abs followed by FDG staining to measure β-galactosidase activity. The overlays show β-galactosidase activity (FDG) in CD4^+^ (top) and CD8^+^ (bottom) T cells after 24 h (left) or 48 h (right) of stimulation. β-galactosidase activity by unstimulated (NS) T cells is shown as negative control. The data are representative of three independent experiments.

Recently, it was shown that ERK3 expression was induced following the activation of the classical MAPKs ERK1/2 in model cell lines [29]. Since ERK1 and ERK2 activation occurs following TCR triggering, we evaluated if ERK1/2 activation was involved in the up-regulation of *Erk3* transcription in activated T cells. To this end, we used the selective pharmacological inhibitor of MEK1/2, U0126, in our T cell activation assay. As shown in Fig. 3, addition of U0126 to anti-CD3 stimulation completely abrogated *Erk3* transcription in both CD4^+^ and CD8^+^ T cells. The same results were obtained with PMA plus ionomycin stimulation (data not shown). Furthermore, a similar reduction in FDG staining was observed in homozygous *Erk3* ^−/−^ T cells, showing that the effect of the inhibitor is independent of ERK3 expression (data not shown). These results show that ERK3 expression is up-regulated via TCR-induced activation of ERK1/ERK2 in CD4^+^ and CD8^+^ T cells.

### Normal Peripheral Lymphoid Compartment in ERK3-deficient Hematopoietic Chimeras

**Figure 4:**
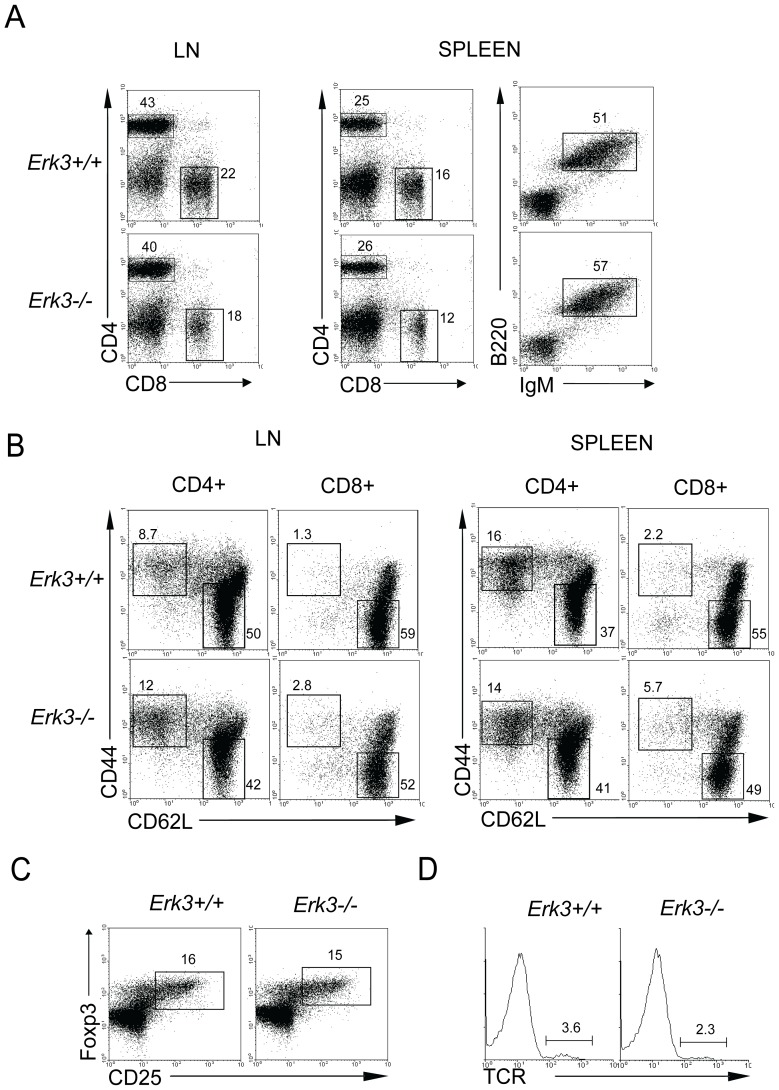
Normal peripheral compartment in ERK3-deficient hematopoietic chimeras. *A*, Lymphocyte subsets distribution in the LN and spleen of hematopoietic chimeras deficient or not for ERK3. CD4/CD8 and B220/IgM profiles are shown for mice reconstituted with *Erk3* ^+/+^ or *Erk*3^−/−^ fetal liver cells. The data are representative of eight independent hematopoietic chimeras. *B*, Phenotype of ERK3-deficient T lymphocytes in the LN and spleen. CD44/CD62L profiles are shown for CD4^+^ and CD8^+^ T cells recovered from the LN and spleen of hematopoietic chimeras deficient or not for ERK3. The data are representative of eight independepent hematopoietic chimeras. *C*, CD4^+^ regulatory T cells are produced normally in the absence of ERK3. CD25/FoxP3 profiles gated on CD4^+^ T cells are shown from the spleen of mice reconstituted with *Erk3* ^+/+^ or *Erk3* ^−/−^ fetal liver cells. *D*, Normal distribution of γδ T cells in the spleen of hematopoietic chimeras deficient or not for ERK3. The histograms show TCRγδ expression gated on CD4^−^CD8^−^CD3^+^ splenocytes. The percentage of TCRγδ^+^ T cells is indicated on the histogram.

The induced expression of ERK3 following T cell activation led us to evaluate if ERK3 was necessary for the response of T cells to anti-CD3 stimulation. We first generated hematopoietic chimeras using *Erk3*-deficient fetal liver cells since *Erk3* ^−/−^ mice die at birth [12]. Unpublished observations from our laboratory support that T cell development occurs in the absence of ERK3 in hematopoietic chimeras (Marquis M et al, in preparation), thus allowing us to study the function of T cells lacking ERK3. As shown in Fig. 4, the lymphoid compartment was normally reconstituted by hematopoietic cells lacking ERK3 expression. CD4^+^, CD8^+^ and B lymphocytes were present in normal percentages (Fig. 4*A*) and numbers (Table 1) in the spleen and LNs. Moreover, the proportion of naive (CD44^lo^ and CD62L^hi^) and activated/memory (CD44^hi^ and CD62L^lo^) T lymphocytes was similar in CD4^+^ and CD8^+^ T cells lacking or not ERK3 (Fig. 4*B*). The generation of regulatory T cells (CD4^+^CD25^+^Foxp3^+^) and γδ T cells was also normal in the absence of ERK3 (Fig. 4*C and D*). Furthermore, *Erk3*-deficient T cells used a normal repertoire of TCR α and β chains as seen using a panel of anti-TCR Vα and Vβ antibodies (Fig. S1). Further analysis did not reveal any difference in the other cell types constituting secondary lymphoid organs when ERK3 is lacking (data not shown).

**Table 1:** Cellularity of secondary lymphoid organs in fetal liver chimeras^a^.

Cell type	Genotype	Spleen (×10^6^)	LN (×10^6^)
CD4^+^ T cells	*Erk3* ^+/+^	31.3±8.8	10.5±3.9
	*Erk3* ^−/−^	30.4±5.2	10.8±2.2
CD8^+^ T cells	*Erk3* ^+/+^	14.7±1.4	4.5±2.8
	*Erk3* ^−/−^	10.9±1.7	3.4±0.8
B cells	*Erk3* ^+/+^	72.1±9.2	ND^b^
	*Erk3* ^−/−^	73.6±9.5	ND

a mean ± SD for at least three experiments.

b not determined.

### Defective T cell Proliferation and Cytokine Production in Absence of ERK3

**Figure 5:**
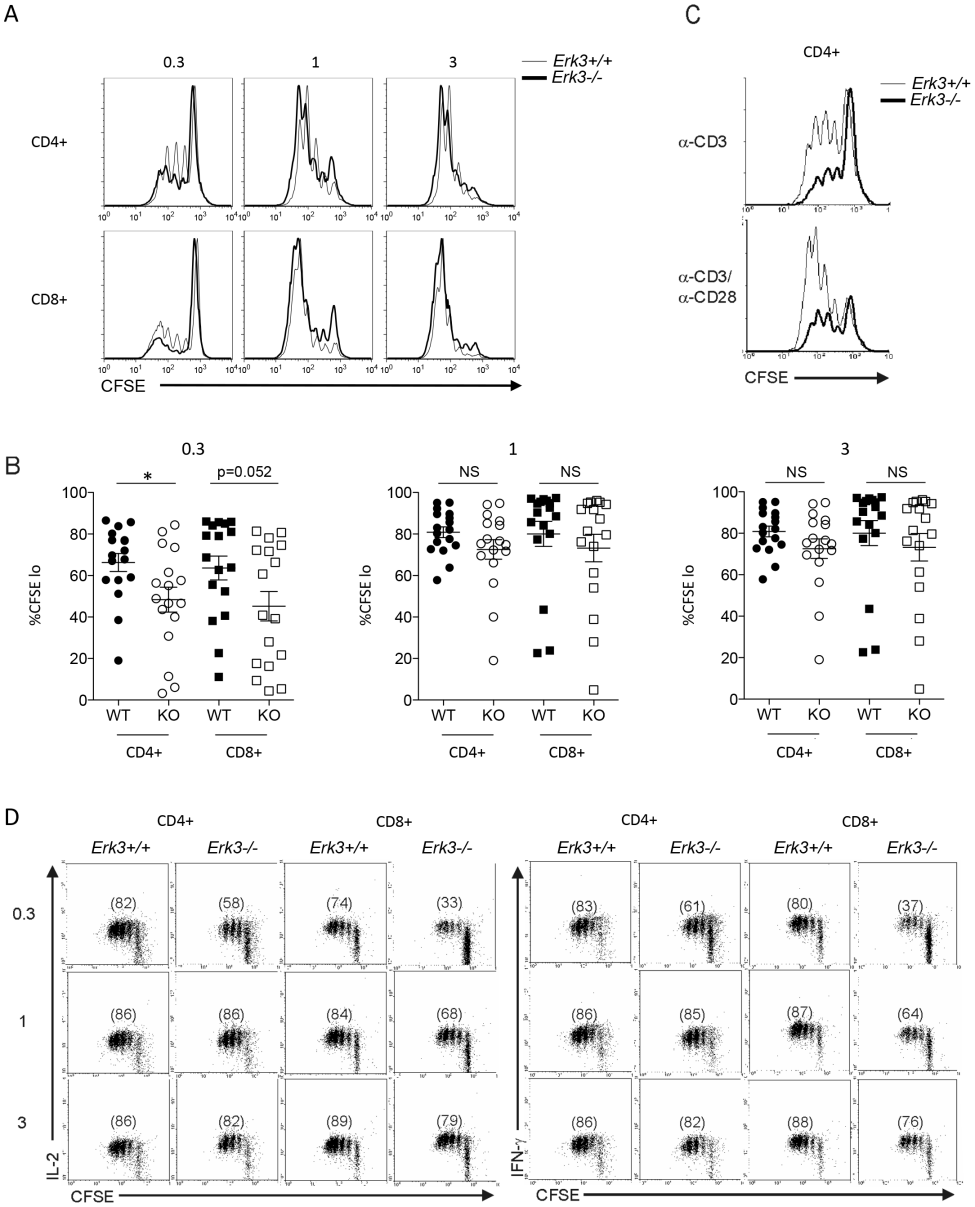
Defective proliferation and cytokine production by ERK3-deficient T cells. *A*, Defective proliferation of *Erk*3^−/−^ T lymphocytes after anti-CD3 stimulation. Splenocytes from *Erk3* ^+/+^ or *Erk3* ^−/−^ hematopoietic chimeras were labeled with CFSE and stimulated with different doses of anti-CD3 Ab for 72 h. CFSE profiles gated on CD4^+^ or CD8^+^ T cells lacking or not ERK3 are shown for the different anti-CD3 Ab concentrations. One representative experiment is shown. *B*, Quantification of T cell proliferation. T cell proliferation, measured as in A, was quantified by determining the percentage of cells that have divided (one division and more; CFSE^lo^). Each dot represents the results from one mouse. Unpaired Student’s t test (two-sided) was used to determine statistical significance. * p<0.05. *C*, Addition of anti-CD28 Abs does not rescue the proliferation of ERK3-deficient CD4^+^ T cells. Splenocytes were stimulated with a sub-optimal dose of anti-CD3 Ab (0.3 µg/ml) in the presence (bottom) or absence (top) of soluble anti-CD28 Ab (5 µg/ml). CFSE profiles gated on CD4^+^ T cells lacking or not ERK3 are shown. *D*, Reduced production of IL-2 and IFN-γ by ERK3-deficient T cells after anti-CD3 stimulation. After 72 h of anti-CD3 stimulation, activated T cells were stimulated with PMA and ionomycin for 4 h. Brefeldin A was added for the last 2 h of culture. IL-2 and IFN-γ production was detected using intracellular cytokine staining. CFSE/IL-2 and CFSE/IFN-γ profiles gated on CD4^+^ or CD8^+^ T lymphocytes deficient or not for ERK3 are shown for the different anti-CD3 Ab concentrations. Numbers in parenthesis represent the % of proliferating and cytokine producing cells. The results in this figure are representative of at least three independent experiments with mice from independent hematopoietic chimeras.

The normal composition of secondary lymphoid organs in absence of ERK3 led us to study if ERK3 participates in T cell activation. *Erk3*-deficient and wild-type splenocytes were labeled with CFSE followed by *in vitro* stimulation with increasing doses of anti-CD3 Abs to evaluate T cell proliferation. As shown in Fig. 5*A–B*, ERK3-deficient CD4^+^ and CD8^+^ T lymphocytes show a reduction in their proliferative capacity when they are stimulated with low dose of anti-CD3 Abs (0.3 µg/ml). The effect reached statistical significance only for CD4^+^ T cells (Fig. 5*B*). Full proliferation of *Erk3* ^−/−^ T cells was restored when high dose of anti-CD3 Abs was used (Fig. 5*A–B*). These results suggest that ERK3 signaling contributes to T cell activation only following weak stimulation. The lack of ERK3 expression by resting T cells suggests that ERK3-induction might be necessary to sustain T cell activation after weak TCR signaling. As expected for a molecule that is induced after stimulation, ERK3 deficiency does not affect the expression of early activation markers such as CD25 and CD69 by T cells (Fig. S2).

To test whether the defective proliferation of ERK3-deficient T cells was intrinsic to the T cells or a consequence of defective co-stimulation provided by APCs, we added soluble anti-CD28 Ab to the anti-CD3 stimulation assay. As shown in Fig. 5*C*, addition of anti-CD28 did not rescue the proliferation of *Erk3* ^−/−^ T cells. This suggests that the reduced T cell proliferation of *Erk3* ^−/−^ splenocytes is probably not due to defective co-stimulation by APCs. These findings argue that the defective proliferation of ERK3-deficient T cells is intrinsic and cannot be compensated by strong co-stimulation.

We then evaluated if the reduced T cell proliferation that we observed with ERK3-deficient T lymphocytes was associated with a reduction in cytokine production by activated T cells. CFSE-labeled splenocytes were stimulated *in vitro* with different concentrations of anti-CD3 antibodies for 72 h. *Erk3* ^−/−^ CD4^+^ and CD8^+^ T cells showed a reduction in the percentage of cells producing IL-2 and IFN-γ when stimulated with low dose of anti-CD3 (0.3 µg/ml) (Fig. 5*D*). Cytokine production by ERK3-deficient T lymphocytes was normal when high dose (3 µg/ml) of anti-CD3 antibodies was used (Fig. 5*D*). However, the quantity of cytokine produced following stimulation with low dose of anti-CD3 was similar in WT and ERK3-deficient T cells. These data indicate that ERK3 expression is necessary for optimal T cell activation.

To address whether the defect in T cell proliferation was due to an increased production of suppressive cytokines, we measured the levels of IL-10 and TGF-β production in wild-type and ERK3-deficient T cells. As shown in Fig. S3 *A*, no difference in IL-10 and TGF-β production was observed between the two groups of T cells. Moreover, *Erk3* ^+/+^ and *Erk3* ^−/−^ T cells produce similar levels of TNF-α (Fig. S3 *A*).

The reduced IL-2 production in ERK3-deficient T cells after anti-CD3 stimulation led us to evaluate if IL-2 supplementation during stimulation would correct the proliferative defect of *Erk3* ^−/−^ T cells. As shown in Fig. S3 *B*, IL-2 addition was able to correct the proliferative defect of *Erk3* ^−/−^ T cells. These results suggest that reduced IL-2 availability may be responsible for the defective proliferation of ERK3-deficient T cells following stimulation with low dose of anti-CD3.

### ERK3-deficient T cells Express Normal Amount of Cyclin D3 and Cdc14A

**Figure 6:**
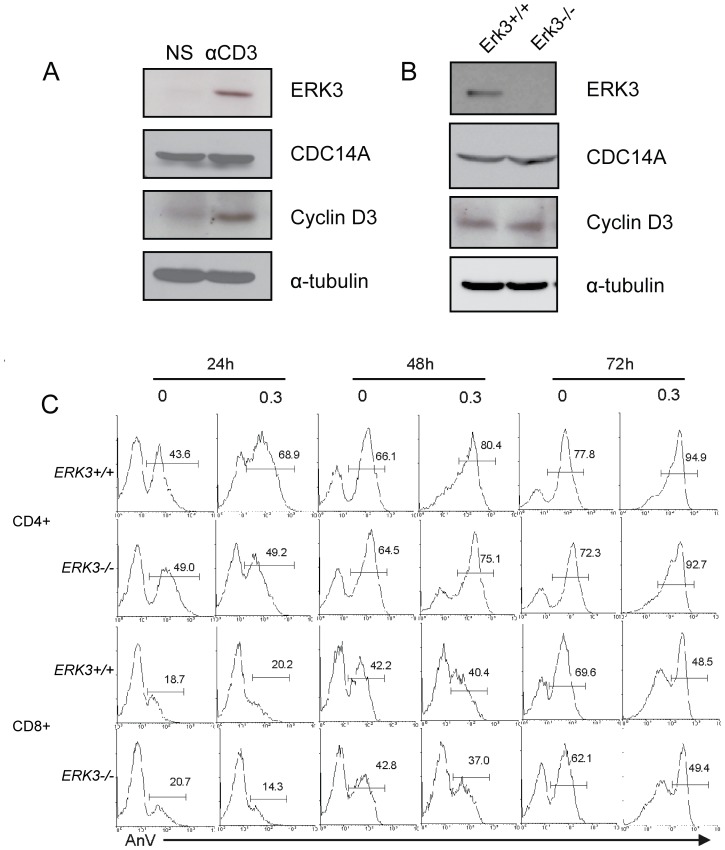
Loss of ERK3 has no effect on cyclin D3 and Cdc14A expression or cell death in ERK3-deficient T cells. *A*–*B*, Cyclin D3 and Cdc14A proteins levels are independent of ERK3 in activated T cells. *A,* Western blot analysis of ERK3, Cdc14A and cyclin D3 proteins in lysates of unstimulated (NS) and anti-CD3 stimulated (α-CD3 3 µg/mL; 72 h) wild-type splenocytes (without T cell purification). Anti-α-tubulin Ab was used as loading control. *B*, Western blot analysis of ERK3, Cdc14A and cyclin D3 proteins in lysates of *Erk3* ^+/+^ and *Erk3* ^−/−^ total splenovytes from hematopoietic chimeras stimulated for 72 h with anti-CD3 Abs (3 µg/mL). Anti-α-tubulin Ab was used as loading control. One representative experiment out of two is shown. *C*, No increase death of ERK3-deficient T cells after anti-CD3 stimulation. Splenocytes from *Erk3* ^+/+^ or *Erk3* ^−/−^ hematopoietic chimeras were stimulated or not with anti-CD3 Ab (0.3 µg/ml). Cells were harvested at different times after activation (24, 48 and 72 h) and stained with AnnexinV (AnV) to measure cell death. AnV profiles gated on CD4^+^ or CD8^+^ T cells lacking or not ERK3 are shown for different time of anti-CD3 stimulation. The results are representative of three independent experiments.

Among the few known ERK3 interacting partners, two are involved in the control of cell cycle progression, namely cyclin D3 and Cdc14A. To test whether defective T cell proliferation in absence of ERK3 is a direct consequence of inappropriate expression of cyclin D3 or Cdc14A, we measured the level of these proteins in activated *Erk3* ^+/+^ and *Erk3* ^−/−^ T cells. The level of cyclin D3 increases following T cell activation while Cdc14A expression remains the same in wild-type splenocytes (Fig. 6*A*). After 72 h of anti-CD3 stimulation, we did not detect any difference in the protein levels of both cyclin D3 and Cdc14A between *Erk3* ^+/+^ and *Erk3* ^−/−^ T cells (Fig. 6*B*). Importantly, no ERK3 protein was detected in T cells coming from *Erk3* ^−/−^ fetal liver chimeras (Fig. 6*B*) confirming ERK3 deficiency in this model. These results suggest that the defective proliferation is not due to improper regulation of cyclin D3 or Cdc14A in ERK3-deficient T lymphocytes.

### ERK3 Deficiency does not Reduce the Survival of Activated T Lymphocytes

To understand how ERK3 affects T cell proliferation and cytokine production we examined if activated T cells were more prone to death when ERK3 was absent. To this end, we performed a kinetic analysis of T cell apoptosis during anti-CD3 stimulation. *Erk3* ^−/−^ and wild-type splenocytes were *in vitro* stimulated with anti-CD3 Abs and were collected at different times after stimulation to monitor apoptosis. The fraction of ERK3-deficient CD4^+^ and CD8^+^ T cells that die by apoptosis (Fig. 6*C*) or necrosis (not shown) was not different from their wild-type counterpart. Moreover, analysis at earlier time points (0 and 12 h) did not reveal any survival difference between *Erk3* ^+/+^ and *Erk3^−/−^* T cells (not shown). This suggests that the decreased proliferation of ERK3-deficient T cells results from a defect in cellular activation that leads to a reduction in the number of cells entering into proliferation following anti-CD3 stimulation.

## Discussion

Our results ascribe a new function for the atypical MAPK ERK3 in T cell activation. We have shown that resting T cells do not express ERK3 but that stimulation of T cells leads to the transcription of the *Erk3* gene. The induction of ERK3 expression is dependent on the activation of the classical MAPKs ERK1 and ERK2, revealing a functional cross-talk between the two MAPK modules. This is in agreement with another report that has shown a role for the ERK1/2 signaling pathway in the up-regulation of ERK3 expression in cell lines [29]. Therefore, the regulation of ERK3 expression is similarly controlled by ERK1/2 signaling in primary cells. The lack of ERK3 expression by resting T cells suggests that constitutive expression could be deleterious to resting T cells. This might be related to the fact that ERK3 is constitutively phosphorylated in its activation loop [9] and thus probably constitutively active when expressed in cells [5], [9]. It is believed that ERK3 activity is mainly regulated by its abundance. In model cell lines, ERK3 is very unstable in highly proliferating cells and its stability increases with cellular differentiation that is coupled with cell cycle arrest [11]. The up-regulation of *Erk3* transcription in T cells after anti-CD3 stimulation is accompanied with an accumulation of the ERK3 protein. Our results also showed that unlike T cells, resting B cells actively transcribed the *Erk3* gene. However, we were not able to detect any ERK3 protein in the spleen which contains a high proportion of B cells (more than 60% of the splenocytes are B cells). This suggests that ERK3 is constitutively degraded in resting B cells. Furthermore, the ERK3 protein in T cells is functional as shown by its phosphorylation on Ser189 and its association with MK5. This indicates a conservation of the ERK3 signaling pathway in primary T cells. The concomitant induction of ERK3 and MK5 protein levels in T cells suggests that these molecules need to work together to mediate their function. Therefore, ERK3 phosphorylation and association with MK5 might be necessary to allow ERK3 function in T lymphocytes. Further studies should reveal whether ERK3 phosphorylation and MK5 association contribute to the effect of ERK3 on T cell proliferation.

Our results suggest an important function for ERK3 induction in T cells to sustain their proliferation after antigenic stimulation. The observation that ERK3 is not expressed by resting T cells but rather induced after stimulation indicates that ERK3 does not participate in early TCR signaling events. This is in agreement with the fact that *Erk3* ^−/−^ T lymphocytes efficiently up-regulate the expression of early activation markers such as CD69 and CD25. Therefore, we hypothesize that ERK3 signaling is needed to sustain T cell activation after initial TCR engagement. This is most important during an *in vivo* immune response since it is well established that sustained T cell activation is required for optimal T cell proliferation and differentiation [30]–[34]. Furthermore, the acquisition of the ability to produce cytokine by T cells is directly related to the number of divisions made by T lymphocytes [35], [36]. Thus, sustained T cell activation dependence on ERK3 may be important to promote the proper acquisition of effector functions by T lymphocytes during an antigenic response.

Interestingly, similar results were recently reported in an ERK2-deficient mouse model [4]. The conditional ablation of ERK2 in T cells leads to a dramatic decrease of anti-CD3 stimulated proliferation of CD8^+^ T cells and to a diminution of IL-2 production [4]. The decrease activation of ERK2-deficient T cells was correlated with decreased expression of Bcl-2 and Bcl-X_L_ and increased Bim expression [4]. Since ERK1/2 activation are responsible for the induction of ERK3 expression in activated T cells, it is tempting to speculate that defective ERK3 induction will occur in absence of ERK2, which then will lead to decreased T cell proliferation. Therefore, we would like to propose that ERK3 represents one of the effector branches of the ERK2 signaling pathway in T cells. In contrast to our results, ERK2-deficiency leads to altered CD69 and CD25 up-regulation after activation of CD8^+^ T cells [4]. This difference can easily be explained by the fact that ERK2 is an early downstream target of TCR signaling suggesting that its activation is necessary for early activation events following TCR stimulation. ERK3-deficiency does not interfere with the expression of early T cell activation markers because its expression is likely induced subsequently to CD69 and CD25 up-regulation. We have also shown that ERK3-deficiency does not decrease T cell proliferation via augmentation of the apoptotic rate of activated T cells. This suggests that ERK3 is required to sustain T cell activation and proliferation but not to promote T cell survival when stimulation is suboptimal. Moreover, the fact that we observed reduced proliferation without an increase in cell death suggests that ERK3 might be influencing the number of cells entering into proliferation. The decrease proliferation rate may also be a consequence of the reduced IL-2 availability following stimulation with low dose of anti-CD3 Abs.

Our results suggest a role for ERK3 when T cells are stimulated with sub-optimal or low concentration of anti-CD3 Abs. This may be significant during *in vivo* response to low level of Ags. Further studies are required to decipher the role of ERK3 during *in vivo* T cell response.

Very few interacting partners of ERK3 have been identified [13], [14], [37]–[39] and their *in vivo* relevance has not been studied yet [5]. Interestingly, two of the molecules that were identified in yeast two-hybrid screens are involved in the control of the cell cycle, cyclin D3 and Cdc14A [37]. Therefore, it was possible that defective T cell proliferation in absence of ERK3 is a direct consequence of inappropriate regulation of cyclin D3 and Cdc14A, which are key regulators of cell cycle progression [40]–[42]. However, we did not observe any difference in the expression levels of cyclin D3 and Cdc14A between *Erk3* ^+/+^ and *Erk3* ^−/−^ T cells. This suggests that the defective T cell proliferation is not a consequence of improper regulation of cyclin D3 and Cdc14A expression in absence of ERK3.

More recently, ERK3 was shown to interact and phosphorylate steroid receptor coactivator 3 [43], a coactivator of nuclear receptors and other transcription factors [44]. However, no role for steroid receptor coactivator 3 in T cell functions has been described yet. Further studies should reveal whether ERK3 acts via this molecule in T cells.

T cell proliferation is not completely abrogated in *Erk3* ^−/−^ T cells suggesting that their might be some redundancy in function with other signaling proteins. We have ruled out that ERK4 has a redundant function in T cell activation since it is not expressed by resting and activated T cells (Fig. S4). This indicates that ERK3 might be the sole molecule controlling MK5 activity in T cells. It is also possible that some T cells can be activated in the absence of ERK3 due to stochastic variation in the level of key signaling molecules by resting T cells [45].

In conclusion, ERK3 plays an important role in sustaining T cell activation to promote proliferation and differentiation. Furthermore, our results also demonstrate that ERK3 is a physiological downstream effector of the ERK1/2 signaling pathway in T cells. The identification of a novel role for ERK3 in T cell activation will allow for a better understanding of the mechanism controlling T cell proliferation, a key event to successfully figth infections and cancers.

## Supporting Information

Figure S1 **Polyclonal repertoire of TCR usage by T cells lacking ERK3.** CD4^+^ and CD8^+^ T cells from the spleen of *Erk3* ^+/+^ and *Erk3* ^−/−^ fetal liver chimeras were stained with a panel of anti-TCR Vβ (A) and anti-TCR Vα (B) Abs. The percentage of Vα^+^ and Vβ^+^ cells within the CD4^+^ and CD8^+^ fractions is shown for *Erk3* ^+/+^ and *Erk3* ^−/−^ T cells.
(DOC)

Figure S2 **(A) Splenocytes from mice reconstituted with** ***Erk3*** **^+/+^ or** ***Erk3*** **^−/−^ fetal liver cells were stimulated with different doses of anti-CD3 Ab (0.3 and 1 µg/ml) or with PMA (50 ng/ml) and ionomycin (500 ng/ml) (P+I) for 24 h.** Cells were harvested and stained with anti-CD4, anti-CD8, anti-CD25 and anti-CD69 Abs.
(DOC)

Figure S3 **(A) Intracellular staining for TNF-α, TGF-β and IL-10 was performed on splenocytes from** ***Erk3*** **^+/+^ and** ***Erk3*** **^−/−^ chimeras following 72 h stimulation with 0.3 µg/ml of α-CD3.** One representative experiment out of 2 is shown. Iso: isotype control. (B) Splenocytes from *Erk3* ^+/+^ and *Erk3* ^−/−^reconstituted fetal liver chimeras were stimulated with 0.3 µg/ml αCD3 for 72 h in the presence or absence of 0.01 µg/ml of rh-IL2. Proliferation was measured by CFSE dilution. One representative experiment out of 2 is shown.
(DOC)

Figure S4 **Lack of transcription of the** ***Erk4*** **gene in resting and activated T cells.** A. No transcription of Erk4 in wild-type T cells. Splenocytes were stimulated with coated anti-CD3 Abs (1 µg/ml) for the indicated time before RNA extraction and RT-PCR analysis of *Erk4* and *Hprt*. Brain RNA was used as a positive control (ctrl). NS, splenocytes that were not stimulated with anti-CD3 Abs. B. No transcription of the *Erk4* gene in *Erk3* ^−/−^ resting and activated T cells. RT-PCR was performed as in A on CD4^+^ or CD8^+^ sorted T cells from *Erk3* ^+/+^ or *Erk3* ^−/−^ hematopoietic chimeras. A reference sample (described in the materials and methods section) was used as a positive control for Erk4 transcription and GAPDH was used as internal control.
(DOC)
