# Correction: The Non-Classical MAP Kinase ERK3 Controls T Cell Activation

**DOI:** 10.1371/journal.pone.0104727

**Published:** 2014-08-01

**Authors:** The PLOS ONE Staff

There are errors in Figure 5B. The same panels have been inserted twice, and the panels for 1μg and 3μg are identical. Please see the corrected Figure 5 here.

**Figure 5:**
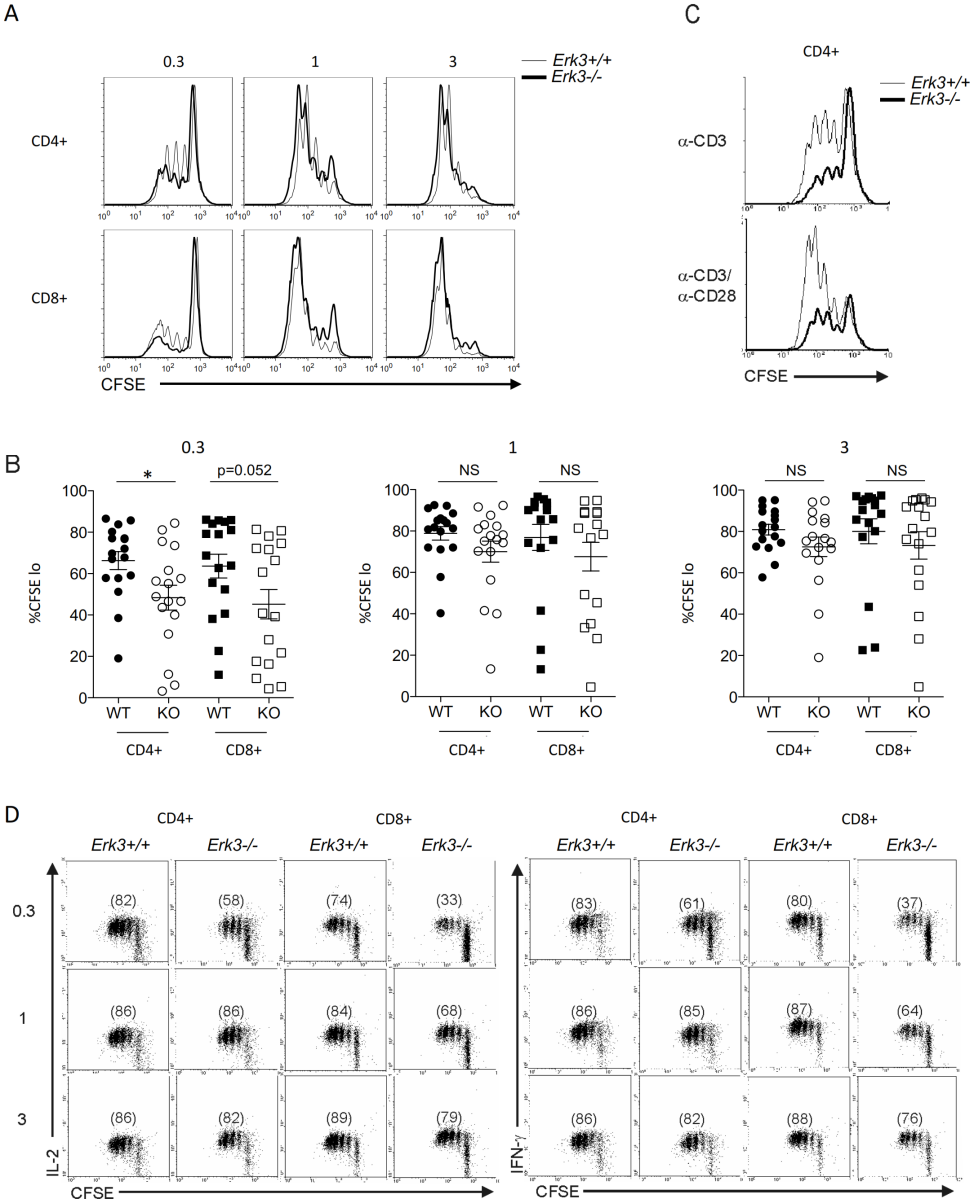
Defective proliferation and cytokine production by ERK3-deficient T cells. *A*, Defectiveproliferation of *Erk*3^−/−^ T lymphocytes after anti-CD3 stimulation. Splenocytes from *Erk3* ^+/+^or *Erk3* ^−/−^ hematopoietic chimeras were labeled with CFSE and stimulated with different doses of anti-CD3 Ab for 72 h. CFSE profiles gated on CD4^+^ or CD8^+^ T cells lacking or not ERK3 are shown for the different anti-CD3 Ab concentrations. One representative experiment is shown. *B*, Quantification of T cell proliferation. T cell proliferation, measured as in A, was quantified by determining the percentage of cells that have divided (one division and more; CFSE^lo^). Each dot represents the results from one mouse. Unpaired Student’s t test (two-sided) was used to determine statistical significance. * p<0.05. *C*, Addition of anti-CD28 Abs does not rescue the proliferation of ERK3-deficient CD4^+^ T cells. Splenocytes were stimulated with a sub-optimal dose of anti-CD3 Ab (0.3 µg/ml) in the presence (bottom) or absence (top) of soluble anti-CD28 Ab (5 µg/ml). CFSE profiles gated on CD4^+^ T cells lacking or not ERK3 are shown. *D*, Reduced production of IL-2 and IFN-γ by ERK3-deficient T cells after anti-CD3 stimulation. After 72 h of anti-CD3 stimulation, activated T cells were stimulated with PMA and ionomycin for 4 h. Brefeldin A was added for the last 2 h of culture. IL-2 and IFN-γ production was detected using intracellular cytokine staining. CFSE/IL-2 and CFSE/IFN-γ profiles gated on CD4^+^ or CD8^+^ T lymphocytes deficient or not for ERK3 are shown for the different anti-CD3 Ab concentrations. Numbers in parenthesis represent the % of proliferating and cytokine producing cells. The results in this figure are representative of at least three independent experiments with mice from independent hematopoietic chimeras
