# Supplementary figures and images for: The Non-Classical MAP Kinase ERK3 Controls T Cell Activation
Source: PLoS One. 2014 Jan 27;9(1):e86681. doi: 10.1371/journal.pone.0086681 (PMC3903551; doi:10.1371/journal.pone.0086681)

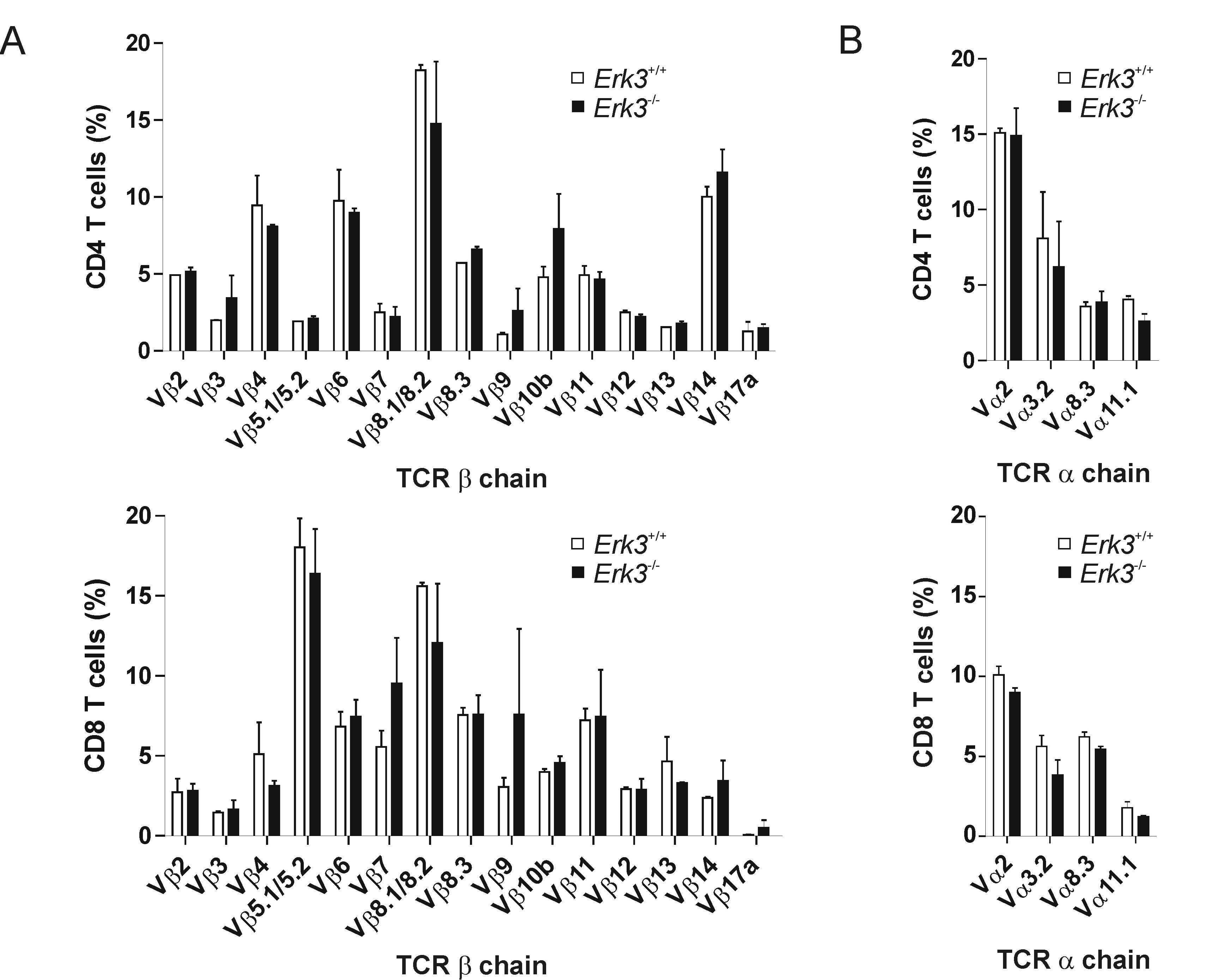

Supplement: Figure S1 — Polyclonal repertoire of TCR usage by T cells lacking ERK3. CD4+ and CD8+ T cells from the spleen of Erk3 +/+ and Erk3 −/− fetal liver chimeras were stained with a panel of anti-TCR Vβ (A) and anti-TCR Vα (B) Abs. The percentage of Vα+ and Vβ+ cells within the CD4+ and CD8+ fractions is shown for Erk3 +/+ and Erk3 −/− T cells. (DOC) [file pone.0086681.s001.doc]

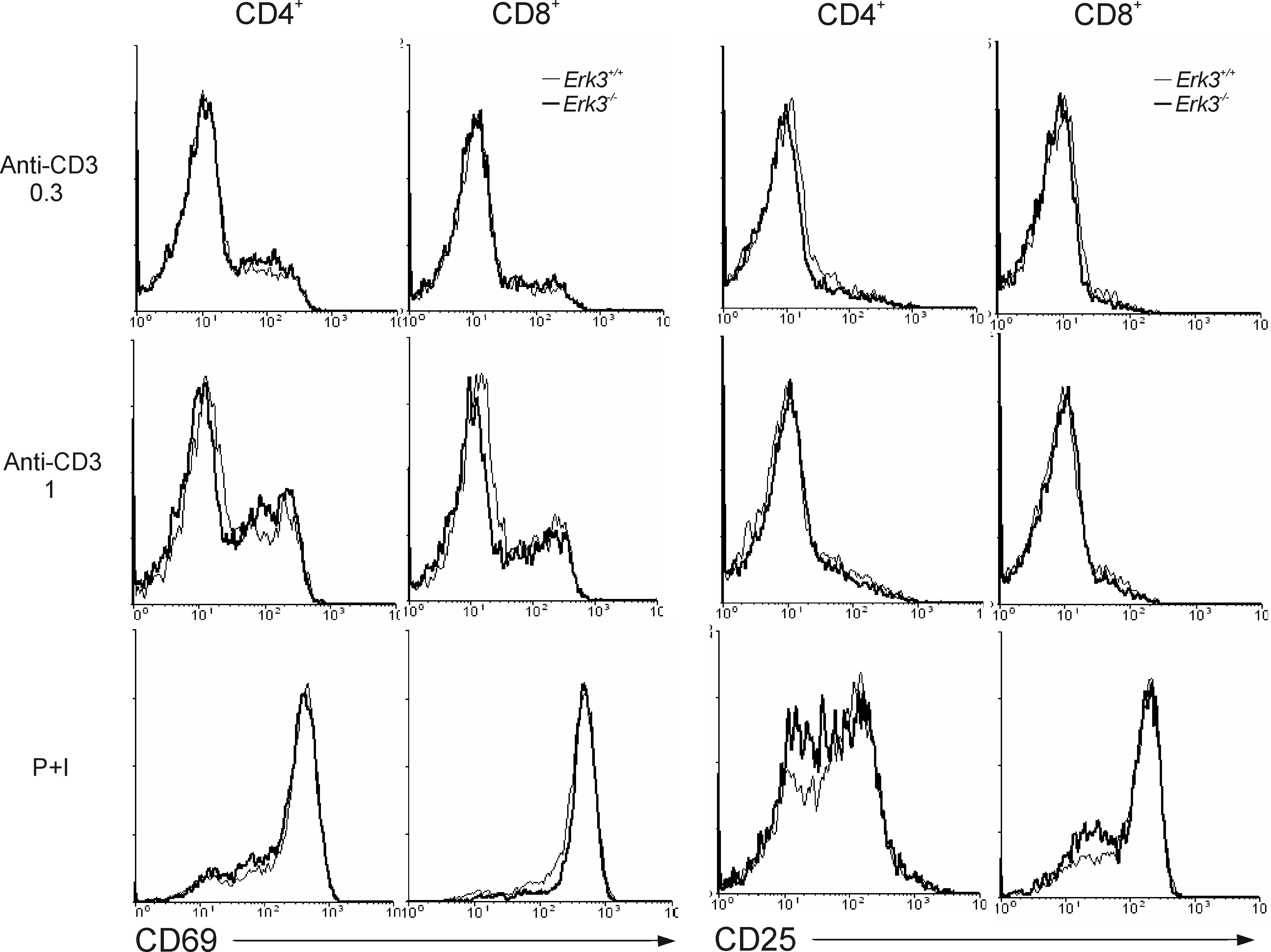

Supplement: Figure S2 — (A) Splenocytes from mice reconstituted with Erk3 +/+ or Erk3 −/− fetal liver cells were stimulated with different doses of anti-CD3 Ab (0.3 and 1 µg/ml) or with PMA (50 ng/ml) and ionomycin (500 ng/ml) (P+I) for 24 h. Cells were harvested and stained with anti-CD4, anti-CD8, anti-CD25 and anti-CD69 Abs. (DOC) [file pone.0086681.s002.doc]

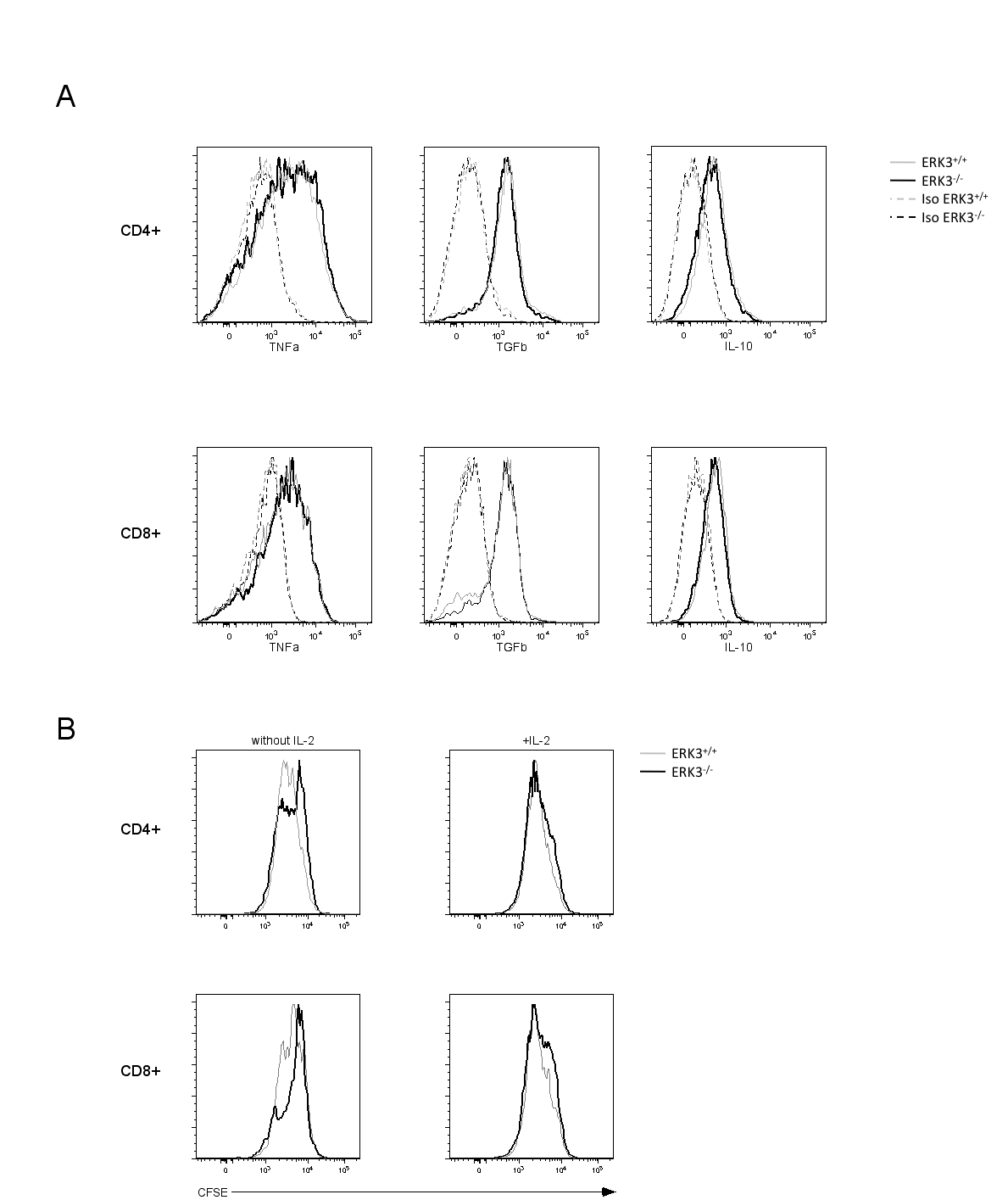

Supplement: Figure S3 — (A) Intracellular staining for TNF-α, TGF-β and IL-10 was performed on splenocytes from Erk3 +/+ and Erk3 −/− chimeras following 72 h stimulation with 0.3 µg/ml of α-CD3. One representative experiment out of 2 is shown. Iso: isotype control. (B) Splenocytes from Erk3 +/+ and Erk3 −/−reconstituted fetal liver chimeras were stimulated with 0.3 µg/ml αCD3 for 72 h in the presence or absence of 0.01 µg/ml of rh-IL2. Proliferation was measured by CFSE dilution. One representative experiment out of 2 is shown. (DOC) [file pone.0086681.s003.doc]

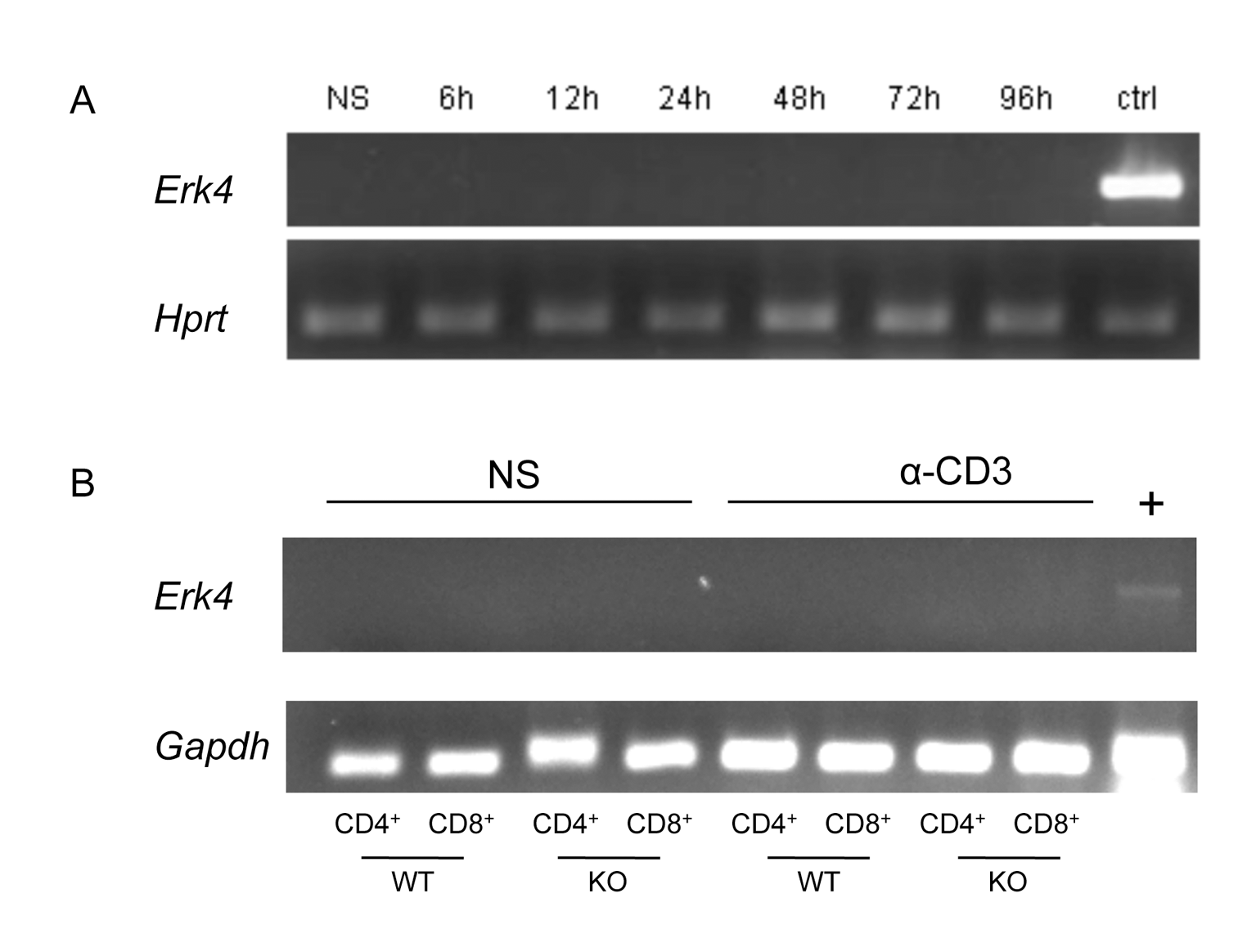

Supplement: Figure S4 — Lack of transcription of the Erk4 gene in resting and activated T cells. A. No transcription of Erk4 in wild-type T cells. Splenocytes were stimulated with coated anti-CD3 Abs (1 µg/ml) for the indicated time before RNA extraction and RT-PCR analysis of Erk4 and Hprt. Brain RNA was used as a positive control (ctrl). NS, splenocytes that were not stimulated with anti-CD3 Abs. B. No transcription of the Erk4 gene in Erk3 −/− resting and activated T cells. RT-PCR was performed as in A on CD4+ or CD8+ sorted T cells from Erk3 +/+ or Erk3 −/− hematopoietic chimeras. A reference sample (described in the materials and methods section) was used as a positive control for Erk4 transcription and GAPDH was used as internal control. (DOC) [file pone.0086681.s004.doc]
